# Association between subtalar articular surface typing and flat foot deformity: which type is more likely to cause flat foot deformity

**DOI:** 10.1186/s12891-021-04872-8

**Published:** 2021-11-23

**Authors:** Lei Zhang, Xiaoyao Peng, Siyuan He, Xin Zhou, Gang Yi, Xiaogao Tang, Bingkun Li, Guoyou Wang, Wanxue Zhao, Yuening Yang

**Affiliations:** 1 Department of Orthopedics, The Affiliated Traditional Chinese Medicine Hospital of Southwest Medical University, Luzhou, 646000 China; 2 Center for Orthopedic Diseases Research, The Affiliated Traditional Chinese Medicine Hospital of Southwest Medical University, Luzhou, 646000 China; 3 Expert Workstation in Luzhou, Luzhou, 646000 China; 4 Clinical Base of The Affiliated Traditional Chinese Medicine Hospital of Southwest Medical University, Guangdong Province Medical 3D Printing Application Transformation Engineering Technology Research Center, Luzhou, 646000 China; 5 School of Clinical Medicine, Southwest Medical University, Luzhou, 646000 China; 6 School of Clinical Medicine, Southern Medical University, Guangzhou, 510000 China

**Keywords:** Flat foot, Meary angle, Subtalar joint

## Abstract

**Background:**

Previous studies have shown a wide range of anatomical classifications of the subtalar joint (STJ) in the population and this is related to the different force line structures of the foot. Different subtalar articular surface morphology may affect the occurrence and development of flat foot deformity, and there are fewer studies in this area. The main objective of our study was to determine the association of different subtalar articular surface with the occurrence and severity of flat foot deformity.

**Methods:**

We analyzed the imaging data of 289 cases of STJ. The articular surface area, Gissane’s angle and Bohler’s angle of subtalar articular surface of different types were counted. The occurrence and severity of flat foot deformity in different subtalar articular surface were judged by measuring the Meary angle of foot.

**Results:**

We classified 289 cases of subtalar articular surface into five types according to the morphology. According to Meary angle, the flat foot deformity of Type I and Type IV are significantly severer than Type II (*P < 0.05*). Type II (7.65 ± 1.38 cm^2^) was significantly smaller than Type I (8.40 ± 1.79 cm^2^) in the total joint facet area(*P < 0.05*). Type III (9.15 ± 1.92 cm^2^) was smaller than Type I (8.40 ± 1.79 cm^2^), II (7.65 ± 1.38 cm^2^) and IV (7.81 ± 1.74 cm^2^) (*P < 0.05*). Type II (28.81 ± 7.44^∘^) was significantly smaller than Type I (30.80 ± 4.61 degrees), and IV (32.25 ± 5.02 degrees) in the Bohler’s angle (*P < 0.05*). Type II (128.49 ± 6.74 degrees) was smaller than Type I (131.58 ± 7.32 degrees), and IV (131.94 ± 5.80 degrees) in the Gissane’s angle (*P < 0.05*).

**Conclusions:**

After being compared and analyzed the measurement of morphological parameters, joint facet area and fusion of subtalar articular surface were closely related to the severity of flat foot deformity and Type I and IV were more likely to develop severer flat foot deformity.

**Level of evidence:**

Level III, retrospective comparative study.

## Background

Flat foot is a deformity of foot caused by various reasons in clinic which is a disease characterized by abduction of forefoot, valgus of the heel and collapse of medial longitudinal arch of foot [1]. At present, the main known pathogenic factors are posterior tibial tendon dysfunction (PTTD), and the occurrence of external trauma such as calcaneus or talus fracture leading to hind foot structural disorder, so that changes in medial longitudinal arch structure and force line can also lead to the occurrence of flat foot [2]. Because the physiological structure and biomechanical composition of the foot are relatively complex, the detection and treatment methods of flat foot need further research in clinic [3, 4]. When the structure of the subtalar joint (STJ) between talus and calcaneus is abnormal, the foot force lines will change and affect the occurrence of ankle joint osteoarthritis [5]. As the most dominant load-bearing joint of the hind foot, the STJ combined with talocalcaneal joint bears weight and transmits to the whole foot, which is very important to maintain the stability of the foot structure [6, 7]. The existing anatomical studies show that the foot arch is mainly composed of transverse arch, lateral longitudinal arch and medial longitudinal arch of foot [8]. The anterior and middle subtalar articular surface of STJ belong to the medial longitudinal arch, and the posterior subtalar articular surface belongs to the lateral longitudinal arch. Therefore, the occurrence of flat foot also involves the change of the anatomical structure of subtalar articular surface. Our research focuses on the relationship between the occurrence and development of flat foot deformity and subtalar articular surface classification.

The study of Kothari et al. proved that the absence of the anterior subtalar articular surface can cause flat foot deformity [9]. When there is a flat foot deformity, the foot is pronated with forefoot abduction, the subtalar joint axis is valgus, and calcaneal valgus results in a change in the force area of the subtalar articular surface. The medially inclined posterior calcaneum articular facets makes the contact force point more concentrated to the lateral part, which may lead to the appearance of lateral subluxation of the STJ [10, 11]. Studies have demonstrated that the assessment of subluxation and discordance in the STJ is an accurate diagnostic tool for acquired flat foot deformity in adults, which may be related to the simple anatomy and small area of the middle subtalar articular surface [12]. And in acquired flat foot deformity, subluxation of the middle articular may better characterize the degree of subluxation of the STJ than that of the posterior STJ [13].

In our previous work, we found that there are extensive anatomical variations of subtalar articular surface in population, which will affect the stress structure of foot and affect the occurrence of osteoarthritis [14]. When the normal anatomical relationship of STJ can be restored, the force line structure of foot can be directly changed and the recovery of midfoot structure can be promoted through the surrounding interosseous ligament, thereby remodels the transverse and longitudinal arches of the foot and relieves a range of symptoms from flat foot [15]. And studies have demonstrated that there is a risk of damage to the subtalar articular surface when performing lateral calcaneal lengthening osteotomy for flat foot without considering the anatomical variation of the STJ [16].

The purpose of this study is to explore the relationship between the different types of subtalar articular surface and the occurrence and severity of flat foot by analyzing the imaging data of STJ, so as to provide the basis for clinical treatment of patients with different types of subtalar articular surface.

## Materials and methods

### Subjects

All procedures were approved by the Ethical Committee of Affiliated Traditional Chinese Medicine Hospital of Southwest Medical University (No. KY2018043). A total of 207 people with 289 STJs and feet met the inclusion criteria (131 male, 76 female), including foot with varying severity of flat foot deformity. There were 159 left calcaneus and foot cases and 130 right calcaneus and foot cases. The average age of patients is 44 (range, 18–80) years. All patients gave informed consent for the experimentation.

Inclusion criteria: (1) Complete development of calcaneus, (2) No history of degenerative disease, fracture, and surgery involving the ankle, especially the subtalar joint, (3) No major systemic disease, tolerable to computed tomography (CT), X-ray examination, (4) The imaging data and other basic information of patients are complete. All patients were screened by medical practitioners and met the above inclusion criteria.

### Methods for classification of subtalar articular surface

The facets morphology of calcaneus were captured by a spiral CT scanner (Siemens AG, Munich, Free State of Bavaria). The images of STJ were reconstructed in three-dimensional (3D) after CT scanning. The STJs were classified according to the number, absence and shape of calcaneal joint surface which was observed by observers. The subtalar articular surface classification was conducted by two medical practitioners. In case of any divergence between the two medical practitioners regarding the results of the classification, a judgement was made by a third medical practitioner. All medical practitioners had more than 5 years of work experience.

Based on previous studies [17], we classified the subtalar articular surface into the following five types:

Type I: the anterior and middle facets of calcaneus fused in a continuous and spindle shape facet, and separated from the posterior facet.

Type II: the anterior, middle, and posterior facets of calcaneus were present independently.

Type III: the anterior facet of calcaneus was absent, and the middle and posterior facets were present independently.

Type IV: the anterior and middle facets of calcaneus fused in a continuous and calabash shape facet, and separated from the posterior facets.

**Fig. 1:**
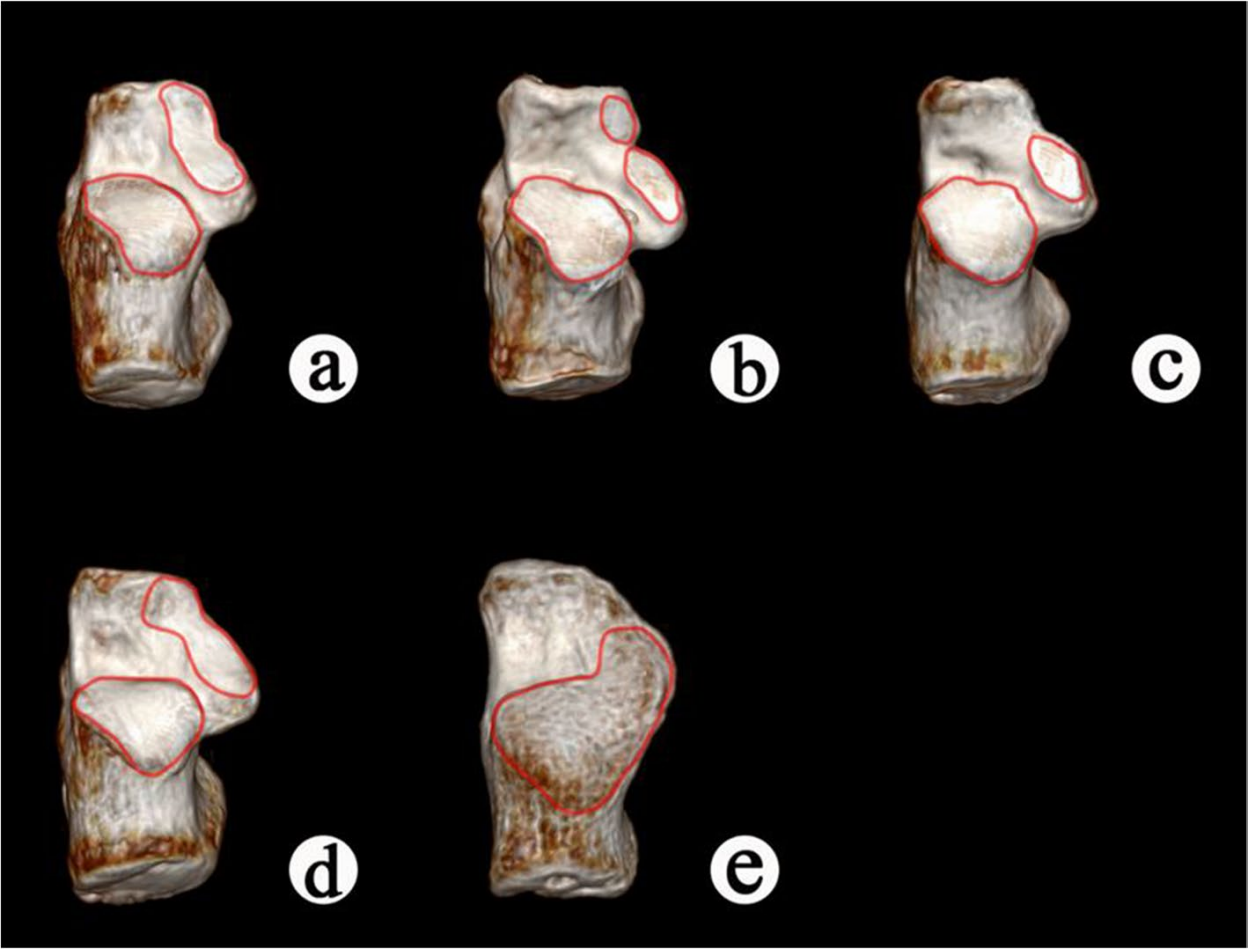
3D reconstruction of subtalar articular surface after CT scanning. **a** Type I, the anterior and middle facets of calcaneus fused in a continuous and spindle shape facet, and separated from the posterior facet. **b** Type II, the anterior, middle, and posterior facets of calcaneus were present independently. **c** Type III, the anterior facet of calcaneus was absent, and the middle and posterior facets were present independently. **d** Type IV, the anterior and middle facets of calcaneus fused in a continuous and calabash shape facet, and separated from the posterior facets. e Type V, the anterior facet of calcaneus was absent, and the middle and posterior facets were fused in a continuous facet

Type V: the anterior facet of calcaneus was absent, and the middle and posterior facets were fused in a continuous facet (Fig. 1).

### The morphological parameters of measurement

**Fig. 2:**
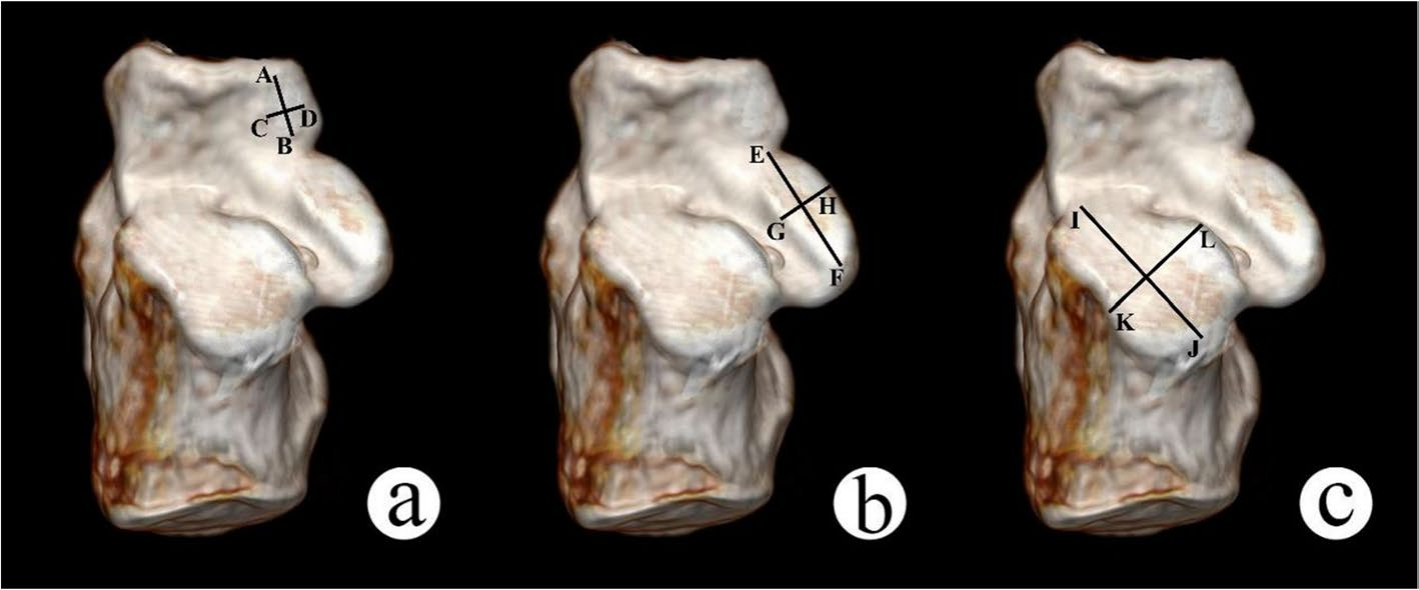
The measurement of total joint facet area. **a** AB: the long-axis of the anterior facet of calcaneus, CD: the short-axis of the anterior facet of calcaneus; **b** EF: the long-axis of the middle facet of calcaneus, GH: the short-axis of the middle facet of calcaneus; **c** IJ: the long-axis of the posterior facet of calcaneus, KL: the short-axis of the posterior facet of calcaneus. Total joint facet area = AB*CD + EF*GH + IJ*KL

As each joint facet is approximated as a rectangle, the joint facet area is calculated by using the short and long axes of each facet. The total joint facet area is obtained by adding up each small facet area (Fig. 2).

The data of angle measurement were from lateral weightbearing radiographs (Siemens AG, Munich, Free State of Bavaria):

Gissane’s angle: The angle is formed by the anterior facet and the posterior facet. When the anterior process joint is missing, it was measured by replacing it with the upper surface of the anterior process of calcaneus.

Bohler’s angle: The angle is measured by the line from the highest point of the posterior facet to the highest point of the anterior process of calcaneus and the line from the upper edge of the calcaneal tubercle to the tangent point of the posterior facet.

Meary angle: The angle is formed by the long axis of the first metatarsal bone (the line between the distal midpoint and proximal midpoint of the first metatarsal diaphysis) and the central axis of talus (the line between the midpoint of the upper and lower surface of the middle talus and the midpoint of the talus neck). When the measurement is complete, the flat foot deformity is graded according to the following criteria (degree) [18]:

Mild: [4,15).

Moderate: [15,30).

Severe: [30, +∞).

### Statistical methods

Categorical variables of the articular facets of the calcaneus were recorded as numbers. All data were presented as the mean and standard deviation (SD). Morphological parameters of measurement of five types of the subtalar articular surface were compared by One-way ANOVA and the statistically significance was considered at *P* ≤ 0.05. Rank sum test was used to compare the severity of flat foot deformity and the statistically significance was considered at *P* ≤ 0.05. The intraclass correlation coefficient (ICC) and 95% confidence interval (95% CI) were used to express the test-retest reliability, and the statistically significance was considered at *P* ≤ 0.05. SPSS version 25.0(IBM Corp, Armonk, NY) for Windows software was used for all statistical analysis.

## Result

We classified the subtalar articular surface into five types based on different characteristics of the calcaneal articular surface morphology, and noted that there were distinct differences in each type: Type I, the anterior and middle facets of calcaneus fused in a continuous and spindle shape facet, and separated from the posterior calcaneus facet; Type II, the anterior, middle, and posterior facets of calcaneus were present independently; Type III, the anterior facet of calcaneus was absent, and the middle and posterior facets were present independently; Type IV, the anterior and middle facets of calcaneus fused in a continuous and calabash shape facet, and separated from the posterior facets; Type V, the anterior calcaneus facet was absent, and the middle and posterior facets were fused in a continuous facet (Fig. 1).

We graded the severity of flat foot by the angle of Meary angle (degree): Mild flat-foot, [4,15); Moderate flat-foot, [15,30); Severe flat-foot, [30,+∞). According to Table 1, of the 143 Type I subtalar articular surfaces, 14 had normal feet, 73 had mild flat foot deformity, 54 had moderate flat foot deformity and 2 had severe flat foot deformity. Of the 31 Type IV subtalar articular surfaces, 3 had normal feet, 19 had mild flat foot deformity, and 9 had moderate flat foot deformity. Of the 61 Type II subtalar articular surfaces, 17 had normal feet, 33 had mild flat foot deformity, and 11 had moderate flat foot deformity, and it had significantly less severity of flat foot deformity than Type I and Type IV (*P < 0.05*) (Table 1). The test-retest reliability of two medical practitioners was excellent: ICC = 0.918[95%CI = 0.898–0.935] (*P* < 0.001).

**Table 1:** Comparison of the severity of flat foot deformity in different types of subtalar articular surface

	No.	Normal	Mild	Moderate	Severe
Type I	143	14	73	54	2
Type II^*^	61	17	33	11	0
Type III	47	12	23	12	0
Type IV^**^	31	3	19	9	0
Type V	7	1	4	2	0

*Note*: ^*^p < 0.05 vs Type I, ^**^p < 0.05 vs Type II. ICC = 0.918[95%CI = 0.898–0.935] (*P* < 0.001)

In terms of total joint facet area, Type II (7.65 ± 1.38 cm^2^) was significantly smaller than Type I (8.40 ± 1.79 cm^2^) (*P < 0.05*). Besides, Type III (9.15 ± 1.92 cm^2^) was significantly smaller than Type I (8.40 ± 1.79 cm^2^), II (7.65 ± 1.38 cm^2^) and IV (7.81 ± 1.74 cm^2^) (*P < 0.05*) (Table 2).

**Table 2:** Calcaneus morphological parameters based on classification

	Average total joint facet area (cm^2^)	Mean Gissane’s angle (degree)	Mean Bohler’s angle (degree)
Type I	8.40 ± 1.79	131.58 ± 7.32	30.80 ± 4.61
Type II	7.65 ± 1.38^*^	128.49 ± 6.74^*^	28.81 ± 7.44^*^
Type III	6.93 ± 1.08^*, **^	130.73 ± 6.36	30.80 ± 4.69
Type IV	7.81 ± 1.74^***^	131.94 ± 5.80^**^	32.25 ± 5.02^**^
Type V	8.18 ± 2.83	130.80 ± 3.73	31.90 ± 7.16

*Note*: Data were presented as the mean and SD. ^*^p < 0.05 vs Type I, ^**^p < 0.05 vs Type II, ^***^p < 0.05 vs Type III

Type II (28.81 ± 7.44 degrees) was significantly smaller than Type I (30.80 ± 4.61 degrees), and IV (32.25 ± 5.02 degrees) in the Bohler’s angle (*P < 0.05*) (Table 2).

Type II (128.49 ± 6.74 degrees) was statistically smaller than Type I (131.58 ± 7.32 degrees), and IV (131.94 ± 5.80 degrees) in the Gissane’s angle(*P < 0.05*) (Table 2).

## Discussion

The etiology and features of flat foot were considered in the original studies to be PTTD, but as research progressed, the widely accepted view was that flat foot was a progressive deformity associated with a variety of structures [19, 20]. The clinical symptoms that manifest as the deformity progresses can also vary and can result from an initial pain in the medial arch affecting a normal gait ultimately progressing to severe conditions such as arthritis due to abnormalities in the force placed on the articular surface [21–24]. Found that the bony support to the talus differed among the different types of the subtalar articular surface, and that stabilization of the STJ when support decreased or when mobility increased would be more dependent on the surrounding ligamentous structures, with extension of time after the ligamentous stretch resulting in decreased static stability of the joint and increased potential pathological activity, giving rise to manifestations of flat foot deformities such as decreased medial longitudinal arch [9, 25, 26].

In our study, we utilized the CT 3D reconstruction technique to measure and type morphological parameters of the subtalar articular surface. In view of the high resolution and 3D reconstruction of Standing (Weightbearing) CT views, it can provide more and accurate measurement parameters for borderline Meary categories compared with Standing (Weightbearing) X-rays, but it still cannot completely replace the X-rays in the process of clinical diagnosis and treatment. All 289 calcaneus were classified into five types according to the articular surface morphological characteristics, and our results showed that the most common was Type I, followed by Type II, III, IV, and V in that order. We indexed the severity of flat foot according to Meary angle in five types of subtalar articular surfaces and found that Type I and IV had significantly higher severity than Type II (*P < 0.05*) (Table 1). Approximately 15% will develop flat foot due to developmental problems, but 7–15% will develop clinical symptoms, meanwhile, the irregular shape of the subtalar articular surface can affect its stability and movement, so the severity of flat foot may be related to the type of subtalar articular surface, while our study showed that Type I and IV were associated with severer flat foot deformity [27, 28].

The key factors affecting the mobility of the STJ are the area of the articular surface and fusion, and a larger and flatter articular surface is associated with greater joint mobility [17, 29]. The total area of the subtalar articular surface was significantly larger in Type I than in Type II (*P < 0.05*) (Table 2). We think that this is because the anterior and middle articular surfaces of the Type I subtalar articular surface were fusing in a spindle shape, while all three articular surfaces of the Type II articular surface existed independently, and this result makes the talus of Type I more mobile relative to the calcaneus and more likely to cause the occurrence of flat foot deformity by causing the alteration of the surrounding ligamentous structures. The triangle formed from three separate articular surfaces according to previous descriptions of Type II constitutes an “osseous tripod” and is therefore best stabilized in all types [26, 30]. The flat foot deformity of Type IV is severer compared with Type II, but there is no obvious difference in the area of the articular surface between these two types, and the reason for this may be because the anterior articular surface and middle articular surface of Type IV are fused in a calabash shape and those of Type I are fused in a spindle shape, but the talus, especially the talus head, of Types I and IV still has a large range of motion, so the degree of flat foot deformity is severer than Type II (*P < 0.05*) (Table 1).

The Gissane’s angle, which is formed by the anterior facet and posterior facet of subtalar articular surface, able to reflect the flatness of the STJ and affect talar mobility [31–33]. Type II was significant sharper Type I and Type IV(*P < 0.05*) (Table 2). The result implies that types I and IV have a greater degree of mobility, thereby affecting the surrounding ligamentous structures and this would be expected to make the flat foot deformity severer in Type I and IV [9, 25].

Our result showed Type II was sharper Type I and Type IV in the Bohler’s angle and is consistent with the severity of the flat foot deformity(*P < 0.05*) (Table 2). Previous studies proved decreased Bohler’s angle was closely related to the increased joint pressure which affects the occurrence of osteoarthritis, we therefore believe that the difference in Bohler’s angle is more likely to be a secondary change rather than a cause of flat foot deformity.

However, this study still had some limitations. (1) Due to the limitation of the study sample size, severe flat foot was found only in Type I and in none of the other types. (2) We will further analyze the association between the differences in left-right flat foot deformity in the same individual and subtalar articular surface typing in our later study. (3) We measured the morphological parameters by performing CT 3D reconstruction of the calcaneus, and this method may produce certain errors.

## Conclusion

After being compared and analyzed the measurement and comparison of morphological parameters, Type I and IV may cause severer flat foot deformity. The area of joint surface area and fusion influenced severity of flat foot deformity by affecting mobility.

## Data Availability

The datasets used and analysed during the current study are available from the corresponding author on reasonable request.

## Abbreviations

STJ: Subtalar joint
PTTD: Posterior tibial tendon dysfunction
CT: Computed tomography
3D: Three-dimensional
SD: Standard deviation
